# Living on the edge: agricultural land use increases genotoxic damage in amphibians on southern brazilian wetlands

**DOI:** 10.1007/s10646-026-03101-z

**Published:** 2026-07-01

**Authors:** Daniela Figueiró, Raquel Fernanda Salla, Guendalina Turcato Oliveira, Camila Fernanda Moser, Renata Krentz Farina, Tainá Ferreira da Silva, Alexandro Marques Tozetti

**Affiliations:** 1 Universidade do Vale do Rio dos Sinos (UNISINOS), Av. Unisinos 950, São Leopoldo, 93022-000 RS Brazil; 2 Universidade Federal de Goiás (UFG), Street 235, Leste Universitário, Goiânia, 74605-050 GO Brazil; 3 Universidade Federal de São Carlos (UFSCar), João Leme dos Santos, Km 110, Sorocaba, 18052-780 SP Brazil; 4 Pontifícia Universidade Católica do Rio Grande do Sul (PUCRS), Av. Ipiranga 6681, Porto Alegre, 90619-900 RS Brazil; 5 Universidade Estadual de Campinas (UNICAMP), Barão Geraldo, Campinas, 13083- 970 SP Brazil

**Keywords:** Micronuclei, Neotropical anurans, Agrochemicals, Pesticides, Mutagenicity, Hematological analysis

## Abstract

Southern Brazil is a key agricultural region in South America, where wetland ecosystems are increasingly impacted by irrigated rice cultivation. Amphibians are particularly sensitive to environmental contamination due to their permeable skin and biphasic life cycle, making them effective bioindicators of ecosystem health. This landscape forms a mosaic of croplands and natural habitats, exposing wildlife to agrochemical contamination. In this study, we evaluated erythrocyte nuclear abnormalities (NAs) in the arboreal amphibian *Dendropsophus sanborni* and the aquatic amphibian *Pseudis minuta*, both inhabiting natural and agricultural wetlands in southern Brazil. Our results revealed significantly higher frequencies of NAs in individuals from agricultural environments across all evaluated biomarkers, indicating a generalized increase in genomic instability. Both micronuclei (MN) and nuclear abnormalities excluding MN (ENAs) increased in agricultural areas, reflecting different dimensions of genotoxic stress. *Pseudis minuta* consistently exhibited higher frequencies of abnormalities than *D. sanborni* and showed stronger responses to habitat type, highlighting the role of ecological traits and exposure pathways in modulating susceptibility to contaminants. Temporal variation was observed for several biomarkers, with higher frequencies occurring during periods coinciding with the onset of agricultural activities. These findings demonstrate that agricultural intensification promotes widespread cellular damage in wetland amphibians and emphasize the importance of integrating multiple biomarkers and species with contrasting ecological traits in biomonitoring programs. Such approaches are essential to improve the detection of sublethal effects of agrochemical exposure and to support conservation strategies in agricultural landscapes.

**Supplementary Information:**

The online version contains supplementary material available at 10.1007/s10646-026-03101-z.

## Introduction

Agricultural expansion is one of the main drivers of environmental degradation worldwide, promoting large-scale land conversion, habitat fragmentation, and the intensification of chemical inputs into natural ecosystems (Foley et al. 2011; Tilman et al. 2011). In Brazil, approximately 33% of the national territory has been converted to agricultural use since the 1980s (MapBiomas, 2022), leading to extensive habitat loss and increased contamination of terrestrial and aquatic environments. In southern Brazil, where irrigated rice and soybean cultivation predominate, agrochemical runoff and leaching have become major sources of surface and groundwater pollution (Pérez-Iglesias et al., 2020; Singh et al., 2022).

These impacts are particularly severe in wetland ecosystems, which are highly sensitive to changes in hydrology and chemical inputs (Davidson et al., 2018). In the Pampas ecoregion, more than 40% of the original landscape has been replaced by agricultural fields (Hasenack et al. 2010; Verdum et al. 2019), resulting in the fragmentation and isolation of natural wetlands that persist as small remnants within agricultural mosaics (Andrade et al. 2023; Tozetti et al. 2023). Although covering only 2.3% of Brazil’s territory, this region supports 62 amphibian species and represents one of the country’s last remaining natural subtropical wetland systems (Borges-Martins et al., 2007; Tozetti et al., 2022).

Within this context, amphibians are among the most affected vertebrates, and are currently recognized as the most threatened group worldwide (IUCN 2025). Many anuran populations have declined due to habitat loss, environmental contamination, and the emergence of infectious diseases (Scheele et al. 2019). Their highly permeable skin and complex life cycles, which involve both aquatic and terrestrial environments, make amphibians especially vulnerable to pollutants (Wells, 2019). Consequently, they are widely regarded as reliable bioindicators of environmental quality.

Southern Brazil is one of the most agriculturally intensive regions in the country, with agrochemical use nearly twice the national average (Cassal et al. 2014). Glyphosate-based herbicides are particularly prevalent and pose significant risks to amphibians due to their permeable skin and strong dependence on aquatic environments (Smalling et al. 2019; Benvindo-Souza et al. 2020). Such exposure has been linked to a wide range of sublethal effects in amphibians, including physiological, developmental, and genetic alterations (Lajmanovich et al. 2014; Pérez-Iglesias et al. 2019).

Among various biomarkers used for environmental monitoring, the micronucleus (MN) test for nuclear abnormalities (NAs) is considered a rapid, sensitive, and cost-effective biomarker (Benvindo-Souza et al. 2025). Micronuclei indicate chromosomal breakage or loss, whereas erythrocytic nuclear abnormalities (ENAs) reflect broader cytological disturbances associated with environmental stress (Josende et al. 2015; Peluso et al. 2020). Although these biomarkers have been widely applied in ecotoxicological studies, most investigations have focused on larval stages under laboratory conditions, leaving the responses of adult anurans in natural landscapes comparatively understudied (Benvindo-Souza et al. 2020).

In this study, we examined erythrocytic nuclear abnormalities in two neotropical anuran species, *Dendropsophus sanborni* and *Pseudis minuta*, inhabiting wetlands embedded in agricultural landscapes of southern Brazil. Specifically, we aimed to (1) determine whether the frequency of nuclear abnormalities differs between wetlands without direct agrochemical application (palm groves) and wetlands subjected to agricultural activities, (2) assess whether abnormality frequency differs between species, and (3) evaluate temporal variation in abnormality frequency across environments and species. We hypothesized that (1) individuals from agricultural areas would exhibit a higher frequency of nuclear abnormalities than those from preserved wetlands and (2) *P. minuta*, a predominantly aquatic species, would display a higher incidence of abnormalities than the arboreal *D. sanborni* due to its greater contact with aquatic environments where agrochemicals tend to accumulate.

## Materials and methods

### Study site

**Fig. 1:**
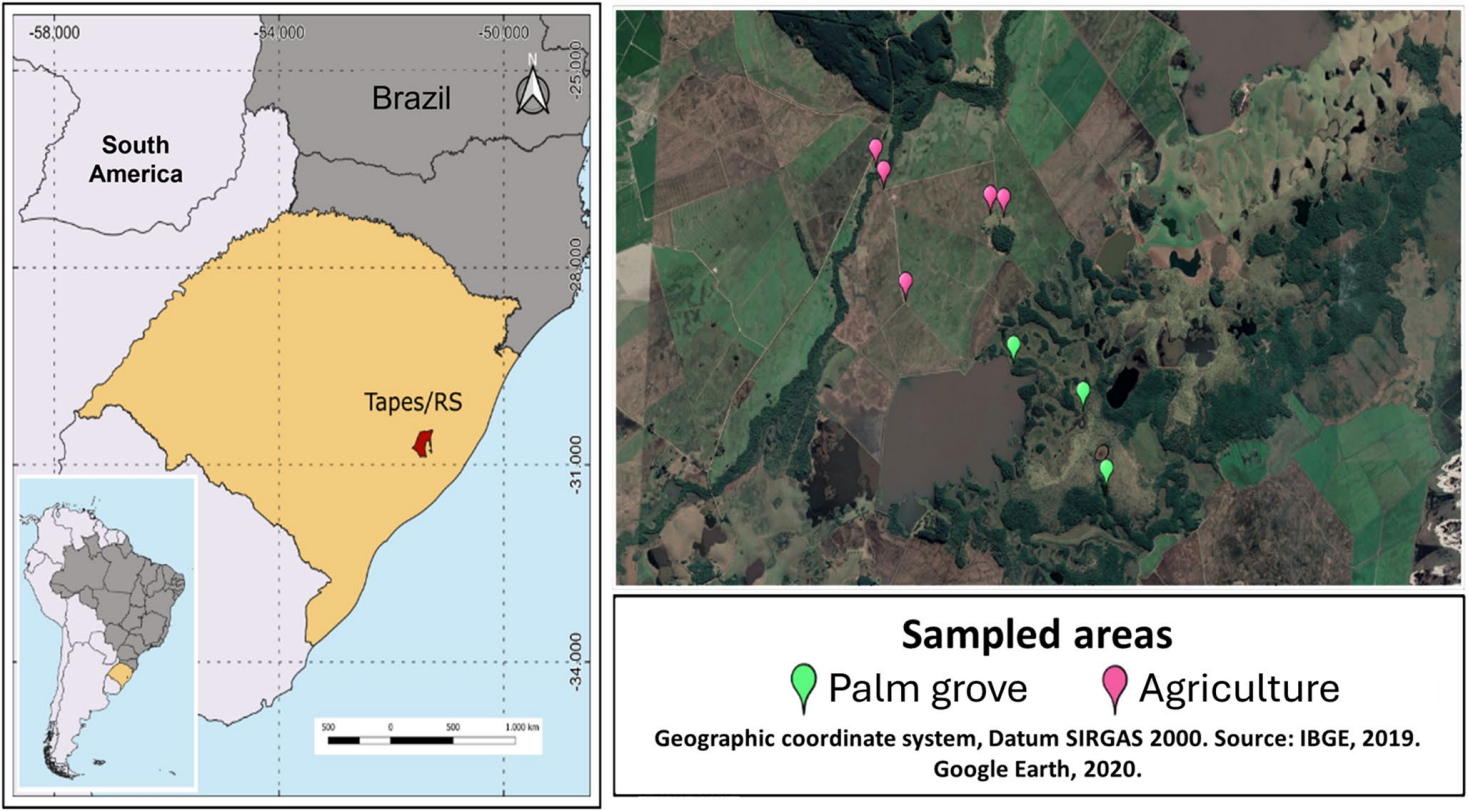
Map illustrating the study area. The first image highlights the municipality of Tapes (red polygon), in the state of Rio Grande do Sul (orange), Brazil (dark gray), while the second image highlights the sampled dots, where the green dots indicate palm grove areas, and the pink dots indicate agricultural environments

Fieldwork was conducted in the municipality of Tapes, southern Brazil (Fig. 1), a region with a subtropical climate, mean annual temperature of 18.8 °C, and annual rainfall of approximately 1213 mm (Maluf 2000). The study took place on a private property (30°31′23.05″S; 51°21′45.08″W) covering 1294.5 ha, located in a transitional zone between the Atlantic Forest and Pampa biomes (Tozetti et al., 2022).

The landscape consists of a mosaic of relatively preserved palm grove habitats (*Butiazal*), dominated by *Butia odorata*, and agricultural areas (Fig. 1). Palm groves occupy approximately 58% of the property (ca. 750 ha) and are not directly subjected to agrochemical application (Verdum et al. 2019). These habitats represent one of the last large remnants of the endangered *Butiazal* ecosystem and include natural wetlands and temporary ponds that serve as important breeding sites for anurans.

The remaining area is used for soybean and irrigated rice cultivation under conventional management (Tozetti et al., 2022), with seasonal agrochemical applications, primarily herbicides based on glyphosate and 2,4-D, along with other compounds used throughout the growing season (Verdum et al. 2019).

Although palm groves are not directly treated with agrochemicals, their proximity to agricultural fields, combined with potential drift, runoff, and hydrological connectivity, suggests that indirect exposure may occur. However, due to lower soil disturbance, greater structural complexity, and predominance of native vegetation, exposure intensity and frequency are expected to be lower than in agricultural areas.

### Biological models and sampling design

Our bioindicator species were adult specimens of the treefrogs *Dendropsophus sanborni* (Sanborn’s Treefrog) and *Pseudis minuta* (Lesser Swimming Frog). These species were selected due to their high local abundance and wide distribution in the study area, allowing consistent sampling across sites and months and ensuring adequate statistical power. In addition, they exhibit contrasting ecological traits: *D. sanborni*: is a small-bodied (15–21 mm), primarily arboreal species associated with shrubs and emergent vegetation, whereas *P. minuta* is a larger (33–56 mm), semi-aquatic species that remains in close contact with water and sediments throughout adulthood (Kwet et al. 2010). These ecological differences are expected to influence exposure pathways and susceptibility to agrochemicals in agricultural landscapes (de Souza et al. 2025).

We sampled eight temporary ponds that serve as amphibian breeding sites (Fig. S1). Sampling at these sites ensures high individual density, reducing spatial and temporal variability, thereby enhancing data quality. To minimize differences between sampling areas, sites were selected based on (a) high densities of both target species and (b) spatial separation to reduce spatial correlations. However, due to logistical constraints such as restricted access during peak flooding and lack of permission to enter certain private areas, the sampling sites were not randomly distributed (Fig. 1 and S1).

### Anuran capture

Sampling was conducted from September to December 2019, a period when adult frogs are more easily located due to their high densities in breeding ponds. A total of 209 individuals were collected, including 109 from agricultural areas (59 *D. sanborni* and 50 *P. minuta*) and 100 from palm grove areas (34 *D. sanborni* and 66 *P. minuta*). Monthly sampling included 32 individuals in September, 75 in October, 77 in November, and 36 in December.

Specimens were actively searched for and manually captured following Heyer et al. (2014). Body mass was measured using a digital scale (precision 0.01 g) and snout–vent length (SVL) with a digital caliper (precision 0.05 mm). Captured individuals were placed in plastic bags, and euthanasia was performed by peritoneal administration of benzocaine (0.25% v/v) until no mechanical response was observed. Blood samples were then immediately collected (see details below). Finally, specimens were preserved in a 10% formaldehyde solution. All procedures complied with Brazilian federal regulations (#70236-2) and were approved by the Ethics Committee on Animal Use of Unisinos (#PPECEUA08.2019).

### Erythrocyte evaluation

Blood samples were collected via cardiac puncture, and a drop of blood was transferred onto glass slides to prepare blood smears (Grisolia and Cordeiro 2000). The smears were preserved by immersion in absolute methanol for 10 min and then stained with Giemsa (10% v/v) for 30 min. For each specimen, two slides were prepared and analyzed, with 1,000 erythrocytes counted per slide under an optical microscope at 1,000x magnification (Bosch et al. 2011; Josende et al. 2015).

Five nuclear abnormalities (NAs) were assessed: micronuclei (MN), kidney-shaped (KS), lobed (LB), notched (NT), and blebbed nuclei (BL) (Fig. S2), which are among the most commonly evaluated abnormalities in similar studies (Bolognesi and Hayashi 2011; Lajmanovich et al. 2014; Corduk et al. 2018). NAs were defined as follows: MN = a small, spherical cytoplasmic body separate from but similar in color to the main nucleus, enclosed by a nuclear membrane; NT = a nuclear region with a slit-like indentation; LB = a nucleus with a protruding rounded fragment; KS = a nucleus with a concave shape resembling a kidney or bean; BL = a nucleus with bubble-shaped protrusions (Fig. S1) (Lajmanovich et al. 2014; Corduk et al. 2018).

While MN represent abnormalities indicative of direct genotoxic effects, the remaining erythrocytic nuclear abnormalities (ENAs) mainly reflect cellular stress and cytotoxicity (Cherednichenko et al. 2024). Thus, the combined analysis of cytogenetic (MN) and cytological (ENAs) markers provides a more comprehensive assessment of environmental contamination and cellular responses to stressors.

All analyses were performed by a single researcher in a blinded, randomized manner using an Olympus optical microscope (1,000x magnification).

### Statistical analysis

The effects of species, environment, and sampling month on the frequency of erythrocytic nuclear abnormalities (NAs) were evaluated using generalized linear models (GLMs) fitted with a negative binomial error distribution and a log link function, implemented in the *glmmTMB* package (Brooks et al. 2023). Separate models were fitted for each biomarker (NAs, ENAs, MN, BL, NT, KS, and LB).

To test for differences among environments, species, and sampling months, models were specified as: *Response* ~ Species + Environment + Month. Species (*Dendropsophus sanborni* and *Pseudis minuta*), Environment (palm grove and agricultural areas), and Month (September–December) were included as fixed effects (Table S1).

To further evaluate species-specific responses to habitat type, models including the interaction between Species and Environment were fitted (*Response* ~ Species * Environment + Month). Estimated marginal means and pairwise contrasts were obtained using the *emmeans* package (Lenth et al., 2019), allowing comparisons between species within each environment and between environments within each species (Tables S2–S3).

When a significant effect of Month was detected (*p* < 0.05), temporal variation was assessed through pairwise comparisons among months using estimated marginal means, with Tukey adjustment for multiple comparisons (Table S4). Additionally, to further evaluate temporal variation within each species, non-parametric Kruskal–Wallis tests were performed separately for *D. sanborni* and *P. minuta*. When significant differences were detected, pairwise comparisons were conducted using Dunn’s test with Holm correction for multiple comparisons (Table S5).

All analyses were conducted in the R environment (R Core Team 2013), and statistical significance was assessed at *p* < 0.05.

## Results

We analyzed a total of 418,000 erythrocytes from 209 specimens, including 109 individuals from agricultural areas (59 *Dendropsophus sanborni* and 50 *Pseudis minuta*) and 100 individuals from palm groves (34 *D. sanborni* and 66 *P. minuta*). Among nuclear abnormalities, micronuclei (MN) were the most frequent, showing higher mean values than erythrocytic nuclear abnormalities excluding MN (ENAs) (MN: *N* = 2,599; x̄ = 11.60 ± 9.40; ENAs: *N* = 1,278; x̄ = 5.90 ± 7.10) (Table 1).

**Table 1:** Mean (± SD) frequency of nuclear abnormalities in adult amphibians from palm grove and agricultural environments in southern Brazil, considering both species combined and separately (*Dendropsophus sanborni* and *Pseudis minuta*)

Biomarker	Palm grove *N* = 100	Agriculture *N* = 109	*D. sanborni* *N* = 93	*P*. minuta *N* = 116
**NAs**	15.4 ± 11.8* (1–44)	19.5 ± 11.9* (2–43)	14.7 ± 11.1 ^a^ (1–44)	19.8 ± 12.2 ^b^ (1–60)
**ENAs**	4.9 ± 5.9* (0–13)	6.9 ± 7.9* (0–18)	4.4 ± 4.5 ^a^ (0–18)	7.2 ± 8.4 ^b^ (0–47)
**MN**	10.5 ± 9.6* (1–44)	12.6 ± 9.1* (0–36)	10.3 ± 9.5 ^a^ (0–44)	12.6 ± 9.2 ^b^ (1–54)
**NT**	2.4 ± 3.3* (0–8)	2.9 ± 3.8* (0–10)	1.9 ± 2.2 ^a^ (0–10)	3.2 ± 4.3 ^b^ (0–24)
**LB**	0.3 ± 0.6* (0–3)	0.5 ± 1.1* (0–6)	0.7 ± 1.1 ^a^ (0–6)	0.9 ± 1.5 ^a^ (0–8)
**KS**	0.3 ± 0.7* (0–3)	0.5 ± 1.1* (0–6)	0.4 ± 1.1 ^a^ (0–6)	0.4 ± 0.7 ^a^ (0–3)
**BL**	1.7 ± 2.5* (0–5)	2.4 ± 3.7* (0–8)	1.3 ± 1.8 ^a^ (0–8)	2.7 ± 3.9 ^b^ (0–21)

NAs = total nuclear abnormalities, ENAs = erythrocytic nuclear abnormalities excluding micronuclei, MN: micronuclei, NT = notched nuclei, LB = lobed nuclei, KS = kidney-shaped nuclei, BL = blebbed nuclei, N = number of individuals. Asterisks (*) indicate significant differences between environments for each biomarker (*p* < 0.05). Different lowercase letters indicate significant interspecific differences in the mean values of each biomarker (*p* < 0.05)

**Table 2:** Nuclear abnormalities in erythrocytes of adult *Dendropsophus sanborni* and *Pseudis minuta* in wetlands of southern Brazil

	*Dendropsophus sanborni*		*Pseudis minuta*	
Biomarker	Palm grove *N* = 34	Agriculture *N* = 59	Palm grove *N* = 66	Agriculture *N* = 50
**NAs**	12.5 ± 10.6 ^a^	15.9 ± 11.4 ^a^	16.9 ± 12.2 ^a, *^	23.7 ± 11.2 ^b, *^
	(1–44)	(2–43)	(1–60)	(3–54)
**ENAs**	3.3 ± 3.5 ^a^	5.1 ± 4.9 ^a^	5.8 ± 6.7 ^b, *^	9.1 ± 9.9 ^b, *^
	(0–13)	(0–18)	(0–32)	(0–47)
**MN**	9.3 ± 9.1 ^a^	10.8 ± 9.7 ^a^	11.1 ± 9.9 ^a, *^	14.6 ± 7.9 ^b, *^
	(1–44)	(0–36)	(0–47)	(0–36)
**NT**	1.7 ± 2.1 ^a^	2.1 ± 2.5 ^a^	2.7 ± 3.2 ^b^	4.3 ± 4.3 ^b^
	(0–8)	(0–10)	(0–16)	(0–20)
**LB**	0.3 ± 0.7 ^a^	0.5 ± 1.3 ^a^	0.3 ± 0.6 ^a^	0.6 ± 0.8 ^a^
	(0–3)	(0–6)	(0–6)	(0–8)
**KS**	0.3 ± 0.7 ^a^	0.5 ± 1.3 ^a^	0.3 ± 0.6 ^a, *^	0.6 ± 0.8 ^a, *^
	(0–3)	(0–6)	(0–3)	(0–2)
**BL**	0.9 ± 1.4 ^a^	1.5 ± 1.9 ^a^	2.0 ± 2.9 ^b^	3.6 ± 4.8 ^b^
	(0–5)	(0–8)	(0–14)	(0–21)

Values are presented as means ± standard error (SE), with minimum and maximum values shown in parentheses. NAs = total nuclear abnormalities, ENAs = erythrocytic nuclear abnormalities excluding micronuclei, MN: micronuclei, NT = notched nuclei, LB = lobed nuclei, KS = kidney-shaped nuclei, BL = blebbed nuclei, N = number of individuals. Different lowercase letters indicate significant interspecific differences within the same environment (*p* < 0.05). Asterisks (*) indicate significant differences between palm grove and agricultural habitats within the same species (*p* < 0.05)

### Environmental influence on nuclear abnormalities

**Fig. 2:**
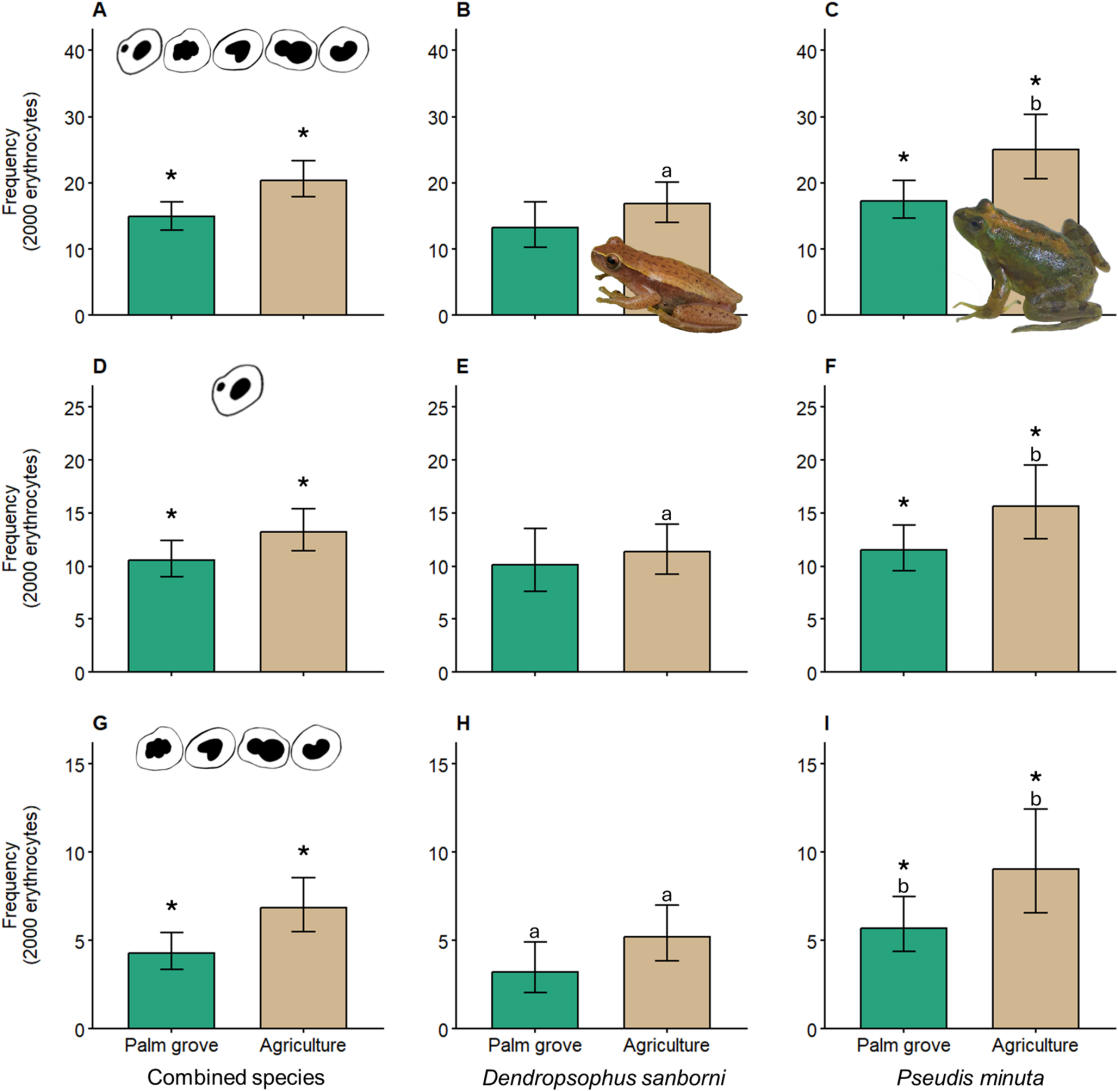
Mean (± SD) frequency of erythrocytic nuclear abnormalities in adult amphibians from wetlands of southern Brazil. Panels represent: (A–C) total nuclear abnormalities (NAs), (D–F) micronuclei (MN), and (G–I) nuclear abnormalities excluding MN (ENAs). First column: both species combined; second column: *Dendropsophus sanborni*; third column: *Pseudis minuta*. Frequencies were calculated based on 2,000 erythrocytes per individual. Asterisks: significant differences between environments (*p* < 0.05). Different lowercase letters: significant interspecific differences within each environment (*p* < 0.05)

**Fig. 3:**
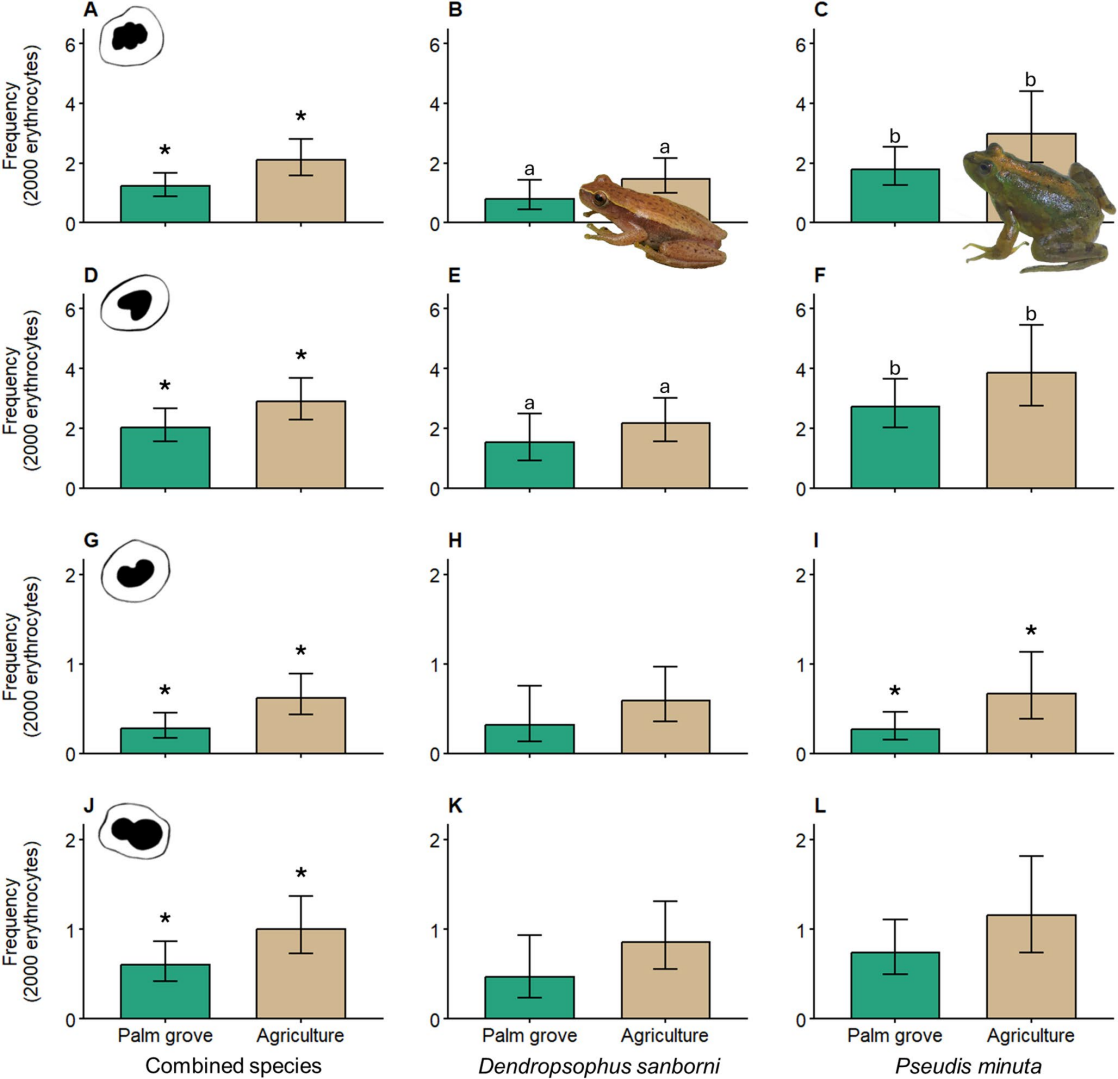
Mean (± SD) frequency of erythrocytic nuclear abnormalities in adult amphibians from palm grove (green) and agricultural (brown) environments. Panels represent: (A–C) blebbed nuclei (BL), (D–F) notched nuclei (NT), (G–I) kidney-shaped nuclei (KS), and (J–L) lobed nuclei (LB). First column: both species combined; second column: *Dendropsophus sanborni*; third column: *Pseudis minuta*. Frequencies were calculated based on 2,000 erythrocytes per individual. Asterisks: significant differences between environments (*p* < 0.05). Different lowercase letters: significant interspecific differences within each environment (*p* < 0.05)

Generalized linear models revealed a significant effect of habitat type on the frequency of nuclear abnormalities (Tables S1; Figs. 2 and 3). Individuals from agricultural environments exhibited higher frequencies across all evaluated biomarkers, including NAs (β = −0.32, *p* < 0.001), MN (β = −0.23, *p* = 0.04), ENAs (β = −0.47, *p* = 0.003), blebbed nuclei (BL: β = −0.55, *p* = 0.01), notched nuclei (NT: β = −0.35, *p* = 0.04), kidney-shaped nuclei (KS: β = −0.79, *p* = 0.01), and lobed nuclei (LB: β = −0.51, *p* = 0.03).

### Species-specific patterns of nuclear abnormalities

Significant interspecific differences were detected for several biomarkers (Table S1; Fig. S3). Compared to *D. sanborni*, *P. minuta* exhibited higher frequencies of NAs (β = 0.34, *p* < 0.001, Fig. S3A), MN (β = 0.23, *p* = 0.03, Fig. S3B), ENAs (β = 0.57, *p* < 0.001, Fig. S3C), BL (β = 0.75, *p* < 0.001, Fig. S3D), and NT (β = 0.58, *p* = 0.001, Fig. S3E). No significant differences were observed for KS (*p* = 0.99) or LB (*p* = 0.13) (Table S1; Fig. S3).

When species were analyzed separately, *P. minuta* exhibited higher frequencies in agricultural habitats for NAs (ratio = 1.45, *p* = 0.004, Fig. 2C), MN (ratio = 1.36, *p* = 0.04, Fig. 2F), ENAs (ratio = 1.59, *p* = 0.03, Fig. 2I), and KS (ratio = 2.49, *p* = 0.02, Fig. 3I) compared to palm groves (Table 2). In contrast, *D. sanborni* did not show significant differences between environments (Table 2; Figs. 2 and 3).

These interspecific differences were consistent across environments (Table S3, Figs. 2 and 3). In agricultural wetlands, *P. minuta* exhibited higher frequencies of NAs (ratio = 0.67, *p* = 0.003, Fig. 2B–C), MN (ratio = 0.73, *p* = 0.04, Fig. 2E–F), ENAs (ratio = 0.57, *p* = 0.01, Fig. 2H–I), BL (ratio = 0.50, *p* = 0.01, Fig. 3B–C), and NT (ratio = 0.56, *p* = 0.02, Fig. 3E–F) compared to *D. sanborni*. In palm grove habitats, *P. minuta* also showed higher frequencies of ENAs (ratio = 0.56, *p* = 0.02, Fig. 2H–I), BL (ratio = 0.45, *p* = 0.02, Fig. 3B–C), and NT (ratio = 0.56, *p* = 0.04, Fig. 3E–F), whereas no significant differences were detected for NAs (*p* = 0.08) or MN (*p* = 0.47). No interspecific differences were observed for KS or LB in either environment (*p* > 0.05).

### Temporal variation in nuclear abnormalities

**Fig. 4:**
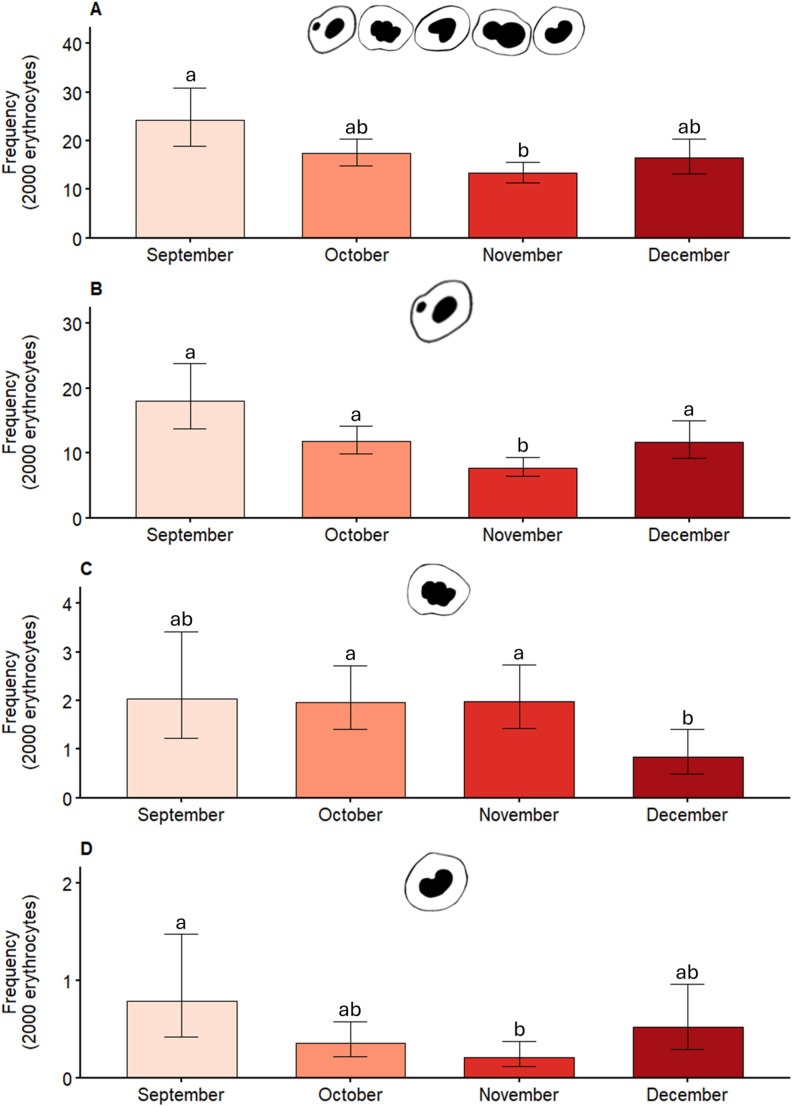
Temporal variation in the mean (± SD) frequency of (A) total erythrocytic nuclear abnormalities (NAs), (B) micronuclei (MN), (C) blebbed nuclei (BL), and (D) kdney-shaped nuclei (KS) from September to December in adult amphibians from palm grove and agricultural environments. Different lowercase letters indicate significant differences among months within each biomarker (*p* < 0.05)

GLM analyses indicated a significant temporal effect for NAs, MN, BL, and KS, whereas no temporal variation was detected for ENAs, NT, or LB (Table S1 and S4, Fig. 4).

Pairwise comparisons revealed that NAs were higher in September compared to November (*p* < 0.001, Fig. 4A). MN were less frequent in November compared to the other months (*p* < 0.01, Fig. 4B). BL were less frequent in December than in October (*p* = 0.03) and November (*p* = 0.03, Fig. 4C). KS were more frequent in September than in November (*p* = 0.01, Fig. 4).

**Fig. 5:**
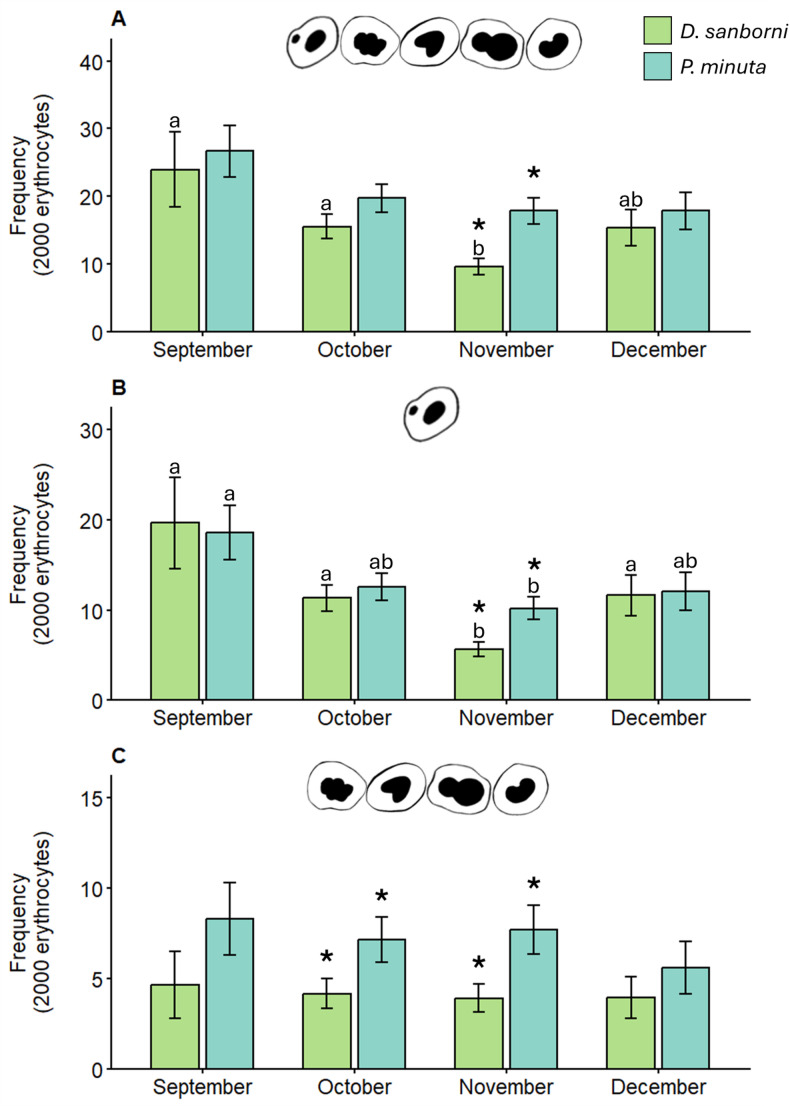
Temporal variation in the mean (± SD) frequency of (A) total nuclear abnormalities (NAs), (B) micronuclei (MN), and (C) nuclear abnormalities excluding MN (ENAs) from September to December in *Dendropsophus sanborni* (green) and *Pseudis minuta* (blue). Frequencies were calculated based on 2,000 erythrocytes per individual. Asterisks indicate significant differences between species within each month (*p* < 0.05), and different lowercase letters indicate significant differences among months within each species (*p* < 0.05)

Significant interspecific temporal differences were also observed (Table S5; Fig. 5). In November, *P. minuta* exhibited higher frequencies of NAs (ratio = 0.54, *p* < 0.001, Fig. 5A), MN (ratio = 0.55, *p* = 0.002, Fig. 5B), ENAs (ratio = 0.51, *p* = 0.01, Fig. 5C), KS (ratio = 0.24, *p* = 0.02), and LB (ratio = 0.27, *p* < 0.01) compared to *D. sanborni* (Table S5). Additionally, BL were higher in *P. minuta* in September (ratio = 0.24, *p* = 0.02) and October (ratio = 0.33, *p* < 0.01), NT in September (ratio = 0.33, *p* = 0.04), and ENAs in October (ratio = 0.58, *p* = 0.04).

## Discussion

Our results demonstrate that agricultural environments are associated with increased frequencies of erythrocytic nuclear abnormalities (NAs) in adult amphibians. Individuals from agricultural areas exhibited higher frequencies across all evaluated biomarkers, including micronuclei (MN), erythrocytic nuclear abnormalities excluding MN (ENAs), blebbed nuclei (BL), notched nuclei (NT), kidney-shaped nuclei (KS), and lobed nuclei (LB), indicating a generalized increase in cellular abnormalities under agricultural influence. This pattern reinforces the relationship between exposure to agrochemicals and genotoxic effects in amphibians, as widely documented in the literature (Lajmanovich et al. 2014; Babini et al. 2016; Mesak et al. 2018; Assis et al. 2022; Pérez-Iglesias et al. 2023).

### Environmental influence on nuclear abnormalities

The frequency of erythrocytic nuclear abnormalities was consistently higher in individuals from agricultural environments across all biomarkers analyzed. Overall, abnormality frequencies were approximately 27% higher in agricultural areas compared to palm groves, with MN increasing by about 25% and ENAs showing a more pronounced increase of approximately 58%. This pattern indicates a broad effect of agricultural activities on genomic integrity.

Similar increases in nuclear abnormalities in agricultural environments have been reported in both in situ and experimental studies ( Pollo et al. 2015; Cruz-Esquivel et al. 2017; Borges et al. 2019), reinforcing the sensitivity of amphibians to land-use changes. The overall increase across all categories suggests that agricultural habitats impose widespread cellular stress rather than affecting isolated biomarkers.

The combined analysis of MN and ENAs provides a comprehensive assessment of genotoxic stress by capturing both chromosomal damage and broader cytological alterations associated with environmental exposure (Cherednichenko et al. 2024). While MN represent chromosomal breakage or loss, ENAs encompass a wider range of nuclear morphological changes related to structural instability (Bolognesi and Hayashi 2011; Benvindo-Souza et al. 2020; Cherednichenko et al. 2024). Together, these markers reflect both accumulated and ongoing genomic damage.

Although agrochemical concentrations were not directly measured and palm grove habitats are located near agricultural fields, some degree of agrochemical transfer may occur between environments through drift, runoff, and hydrological connectivity (Josende et al. 2015). Therefore, palm grove areas should not be interpreted as contamination-free sites, but rather as environments with lower exposure intensity. This interpretation is further supported by studies conducted in similar regions showing physiological and biochemical alterations in amphibians exposed to agricultural contaminants (de Souza et al. 2025; Hentges et al. 2025).

### Species-specific susceptibility and ecological exposure

Species-specific analyses indicate that ecological traits play a key role in modulating genotoxic responses. Regardless of environment, *Pseudis minuta* exhibited higher frequencies of nuclear abnormalities than *Dendropsophus sanborni* across multiple biomarkers, including NAs, MN, ENAs, BL, and NT. In addition, abnormality frequencies in *P. minuta* were approximately 50% higher in agricultural environments, whereas *D. sanborni* did not show significant differences between environments. Higher ENA frequencies in *P. minuta* were also observed in palm grove habitats.

Increased ENA frequencies may reflect not only cytotoxic effects, but also active cellular responses involved in the recognition and elimination of genetically damaged cells (Ray et al., 2005). These abnormalities can therefore represent both damage expression and cellular mechanisms aimed at maintaining genomic integrity under environmental stress (Ray et al., 2005; Fernandes et al.,^2007^).

In particular, *P. minuta* exhibited higher frequencies of blebbed (BL) and notched nuclei (NT) than *D. sanborni* across the two environments evaluated. Although both abnormalities indicate genomic instability, they differ in their underlying mechanisms. Notched nuclei are associated with structural deformation of the nuclear envelope, reflecting invagination processes, whereas blebbed nuclei result from chromatin extrusion and are linked to active genomic stress responses (Cherednichenko et al. 2024). This distinction suggests that BL may represent a more dynamic cellular response to DNA damage, while NT reflects structural alterations in nuclear organization.

These patterns are consistent with differences in habitat use between species. *Pseudis minuta* is predominantly aquatic and maintains continuous contact with water and sediments throughout adulthood, whereas *D. sanborni* has a mainly arboreal habit, with reduced interaction with aquatic environments after metamorphosis (Kwet et al. 2010; Huckembeck et al. 2012; Zaracho et al. 2012). In aquatic systems influenced by agricultural runoff, contaminants such as pesticides and fertilizers are continuously introduced into water bodies, resulting in prolonged exposure of semi-aquatic species (Josende et al. 2015). Under these conditions, the continuous contact of *P. minuta* with water and sediments likely increases exposure to persistent contaminant loads compared to *D. sanborni*.

This chronic exposure may promote sustained nuclear budding activity, reflecting transient genomic instability and cellular attempts to eliminate damaged chromatin prior to micronucleus formation ( Fenech et al., 2011; Kuzina et al., 2018; Pérez-Iglesias et al. 2019). The predominance of BL in *P. minuta* therefore suggests that this species experiences sustained genomic stress and activates nuclear defense mechanisms that may or may not culminate in micronucleus formation.

### Temporal variability and exposure dynamics

Temporal variation was observed for several biomarkers, including NAs, MN, BL, and KS, whereas ENAs, NT, and LB did not show significant variation across months. This pattern indicates that different categories of nuclear abnormalities respond distinctly to temporal fluctuations in environmental conditions.

Among the evaluated biomarkers, MN and KS showed more pronounced temporal variation, with higher frequencies recorded in late winter and early spring, particularly in September. This period coincides with the onset and intensification of agrochemical applications in soybean and irrigated rice systems in southern Brazil (Verdum et al. 2019; Embrapa 2021; Tozetti et al., 2022). During these months, increased runoff and direct application likely elevate contaminant availability in aquatic environments (Cassal et al. 2014; Singh et al. 2022). Similar seasonal trends in genotoxic biomarkers have been reported in amphibians exposed to fluctuating environmental stressors (Lajmanovich et al. 2018), supporting the influence of agricultural practices on these temporal patterns.

These results suggest that MN are more sensitive to short-term exposure pulses, reflecting acute and transient genotoxic effects associated with temporal variation in contaminant input, climatic conditions, and physiological factors such as reproductive activity and metabolic demand (Campana et al. 2003; Brühl et al. 2013; Alnoaimi et al. 2021). In contrast, ENAs appear to reflect more stable or cumulative genomic stress, as indicated by the absence of temporal variation.

The reduction in blebbed nuclei (BL) frequencies observed in December may indicate a decrease in genotoxic pressure or partial cellular recovery following earlier exposure peaks (Fenech et al., 2011; Pérez-Iglesias et al. 2019). Within this context, the period between September and December likely represents a critical window of intensified environmental exposure in agricultural landscapes, with populations experiencing varying levels of contaminant input over time.

## Conclusions

Our findings demonstrate that anurans inhabiting agricultural landscapes in southern Brazil exhibit increased frequencies of erythrocytic nuclear abnormalities across all evaluated biomarkers, including MN, ENAs, and other nuclear alterations, compared to populations from palm grove habitats. This generalized increase indicates that agricultural environments promote widespread genomic instability rather than affecting isolated categories of cellular damage.

The combined use of MN and ENAs proved effective for detecting different dimensions of genotoxic stress, with MN reflecting acute chromosomal damage and ENAs capturing broader and more persistent cytological alterations. Additionally, species-specific patterns highlight the role of ecological traits in modulating exposure and susceptibility, as the aquatic species *Pseudis minuta* consistently exhibited higher frequencies of abnormalities than the more arboreal *Dendropsophus sanborni*. These differences reinforce the importance of water and sediments as key pathways of contaminant exposure in agricultural wetlands.

Together, these results emphasize the sensitivity of amphibians to agricultural intensification and support the use of erythrocytic nuclear abnormalities as reliable biomarkers in environmental monitoring. Reducing agrochemical inputs and improving management practices are essential to mitigate their impacts on amphibian populations and wetland ecosystems. Future studies should prioritize the quantification of agrochemical residues in environmental matrices and biological tissues, as well as investigate the cellular mechanisms underlying different nuclear abnormalities. Expanding comparative approaches across species with distinct ecological traits may further improve the application of these biomarkers in ecological risk assessment and conservation planning in agroecosystems.

## Supplementary Information

Below is the link to the electronic supplementary material.

Supplementary Material 1

## Data Availability

No datasets were generated or analysed during the current study.
