# Supplementary material for: Living on the edge: agricultural land use increases genotoxic damage in amphibians on southern brazilian wetlands
Source: Ecotoxicology. 2026 Jul 1;35(5):127. doi: 10.1007/s10646-026-03101-z (PMC13323865; doi:10.1007/s10646-026-03101-z)
Supplement: Supplementary file 1 — Supplementary Material 1 [file 10646_2026_3101_MOESM1_ESM.docx]

**Table S1.** Results of generalized linear models (GLMs) fitted with a negative binomial distribution to evaluate the effects of species, environment, and sampling month on erythrocytic nuclear abnormalities in adult *Dendropsophus sanborni* and *Pseudis minuta*. Values are presented as estimates ± standard error (SE). NAs = total nuclear abnormalities, ENAs = erythrocytic nuclear abnormalities excluding micronuclei, MN = micronuclei, BL = blebbed nuclei, NT = notched nuclei, KS = kidney-shaped nuclei. Reference levels were *D. sanborni*, agricultural environment, and December. Significant effects are indicated in bold (*p* < 0.05).

| **Biomarker** | **Predictor** | **Estimate** | **SE** | **z value** | ***p*-value** |
| --- | --- | --- | --- | --- | --- |
| **NAs** | Intercept | 2.788 | 0.129 | 21.62 | **<0.001** |
|  | Species (*P. minuta*) | 0.339 | 0.096 | 3.52 | **<0.001** |
|  | Environment (Palm grove) | -0.316 | 0.096 | -3.29 | **0.001** |
|  | November | -0.209 | 0.138 | -1.52 | 0.129 |
|  | October | 0.059 | 0.137 | 0.43 | 0.665 |
|  | September | 0.386 | 0.167 | 2.3 | **0.021** |
| **ENAs** | Intercept | 1.487 | 0.216 | 6.9 | **<0.001** |
|  | Species (*P. minuta*) | 0.565 | 0.16 | 3.53 | **<0.001** |
|  | Environment (Palm grove) | -0.472 | 0.159 | -2.97 | **0.003** |
|  | November | 0.171 | 0.228 | 0.75 | 0.454 |
|  | October | 0.157 | 0.229 | 0.69 | 0.492 |
|  | September | 0.299 | 0.28 | 1.07 | 0.286 |
| **MN** | Intercept | 2.46 | 0.146 | 16.85 | **<0.001** |
|  | Species (*P. minuta*) | 0.234 | 0.11 | 2.13 | **0.033** |
|  | Environment (Palm grove) | -0.226 | 0.109 | -2.07 | **0.039** |
|  | November | -0.415 | 0.157 | -2.65 | **0.008** |
|  | October | 0.009 | 0.155 | 0.06 | 0.952 |
|  | September | 0.43 | 0.189 | 2.28 | **0.023** |
| **BL** | Intercept | -0.277 | 0.303 | -0.92 | 0.36 |
|  | Species (*P. minuta*) | 0.745 | 0.205 | 3.64 | **<0.001** |
|  | Environment (Palm grove) | -0.546 | 0.202 | -2.7 | **0.007** |
|  | November | 0.86 | 0.313 | 2.75 | **0.006** |
|  | October | 0.851 | 0.314 | 2.71 | **0.007** |
|  | September | 0.892 | 0.372 | 2.4 | **0.017** |
| **NT** | Intercept | 0.75 | 0.237 | 3.17 | **0.002** |
|  | Species (*P. minuta*) | 0.578 | 0.177 | 3.26 | **0.001** |
|  | Environment (Palm grove) | -0.353 | 0.175 | -2.02 | **0.044** |
|  | November | 0.094 | 0.249 | 0.377 | 0.706 |
|  | October | -0.032 | 0.251 | -0.129 | 0.897 |
|  | September | 0.044 | 0.307 | 0.143 | 0.886 |
| **KS** | Intercept | -0.251 | 0.344 | -0.73 | 0.466 |
|  | Species (*P. minuta*) | 0.003 | 0.294 | 0.013 | 0.989 |
|  | Environment (Palm grove) | -0.786 | 0.302 | -2.6 | **0.009** |
|  | November | -0.906 | 0.415 | -2.18 | **0.029** |
|  | October | -0.401 | 0.393 | -1.019 | 0.308 |
|  | September | 0.406 | 0.442 | 0.917 | 0.359 |
| **LB** | Intercept | -0.03 | 0.305 | -0.1 | 0.922 |
|  | Species (*P. minuta*) | 0.357 | 0.236 | 1.510 | 0.131 |
|  | Environment (Palm grove) | -0.505 | 0.236 | -2.14 | **0.032** |
|  | November | -0.208 | 0.327 | -0.635 | 0.525 |
|  | October | -1.141 | 0.327 | -0.433 | 0.665 |
|  | September | 0.251 | 0.411 | -0.609 | 0.524 |

**Table S2.** Comparisons between environments within each species. Estimated marginal means (± SE) and pairwise comparisons between environments within each species. Values are on response scale. NAs = total nuclear abnormalities, ENAs = erythrocytic nuclear abnormalities excluding micronuclei, MN = micronuclei, BL = blebbed nuclei, NT = notched nuclei, KS = kidney-shaped nuclei. Significant comparisons (*p* < 0.05) are shown in bold.

| **Biomarker** | **Environment** | **Agriculture** | **Palm grove** | **Ratio** | ***p*-value** |
| --- | --- | --- | --- | --- | --- |
| **NAs** | *D. sanborni* | 16.8 ± 1.54 | 13.2 ± 1.73 | 1.27 | 0.131 |
|  | *P. minuta* | 25.0 ± 2.47 | 17.3 ± 1.43 | 1.45 | **0.004** |
| **ENAs** | *D. sanborni* | 5.20 ± 0.79 | 3.20 ± 0.71 | 1.63 | 0.069 |
|  | *P. minuta* | 9.05 ± 1.47 | 5.71 ± 0.78 | 1.59 | **0.029** |
| **MN** | *D. sanborni* | 11.4 ± 1.18 | 10.1 ± 1.50 | 1.12 | 0.526 |
|  | *P. minuta* | 15.6 ± 1.76 | 11.5 ± 1.09 | 1.36 | **0.037** |
| **BL** | *D. sanborni* | 1.48 ± 0.29 | 0.81 ± 0.24 | 1.83 | 0.083 |
|  | *P. minuta* | 2.99 ± 0.60 | 1.80 ± 0.32 | 1.66 | 0.052 |
| **NT** | *D. sanborni* | 2.18 ± 0.37 | 1.53 ± 0.38 | 1.43 | 0.237 |
|  | *P. minuta* | 3.87 ± 0.68 | 2.72 ± 0.41 | 1.42 | 0.126 |
| **KS** | *D. sanborni* | 0.59 ± 0.15 | 0.32 ± 0.14 | 1.83 | 0.238 |
|  | *P. minuta* | 0.66 ± 0.18 | 0.27 ± 0.08 | 2.49 | **0.023** |
| **LB** | *D. sanborni* | 0.86 ± 0.19 | 0.47 ± 0.17 | 1.82 | 0.148 |
|  | *P. minuta* | 1.16 ± 0.27 | 0.74 ± 0.15 | 1.57 | 0.142 |

**Table S3.** Comparisons between species within each environment. Estimated marginal means (± SE) and pairwise comparisons between species within each environment. Values are on response scale. NAs = total nuclear abnormalities, ENAs = erythrocytic nuclear abnormalities excluding micronuclei, MN = micronuclei, BL = blebbed nuclei, NT = notched nuclei, KS = kidney-shaped nuclei. Significant comparisons (*p* < 0.05) are shown in bold.

| **Biomarker** | **Environment** | ***D. sanborni*** | ***P. minuta*** | **Ratio** | ***p*-value** |
| --- | --- | --- | --- | --- | --- |
| **NAs** | Agriculture | 16.8 ± 1.54 | 25.0 ± 2.47 | 0.673 | **0.003** |
|  | Palm grove | 13.2 ± 1.73 | 17.3 ± 1.43 | 0.766 | 0.084 |
| **ENAs** | Agriculture | 5.20 ± 0.79 | 9.05 ± 1.47 | 0.574 | **0.012** |
|  | Palm grove | 3.20 ± 0.71 | 5.71 ± 0.78 | 0.56 | **0.025** |
| **MN** | Agriculture | 11.4 ± 1.18 | 15.6 ± 1.76 | 0.727 | **0.037** |
|  | Palm grove | 10.1 ± 1.50 | 11.5 ± 1.09 | 0.881 | 0.469 |
| **BL** | Agriculture | 1.48 ± 0.29 | 2.99 ± 0.60 | 0.495 | **0.01** |
|  | Palm grove | 0.81 ± 0.24 | 1.80 ± 0.32 | 0.449 | **0.018** |
| **NT** | Agriculture | 2.18 ± 0.37 | 3.87 ± 0.68 | 0.562 | **0.017** |
|  | Palm grove | 1.53 ± 0.38 | 2.72 ± 0.41 | 0.56 | **0.045** |
| **KS** | Agriculture | 0.59 ± 0.15 | 0.66 ± 0.18 | 0.892 | 0.764 |
|  | Palm grove | 0.32 ± 0.14 | 0.27 ± 0.08 | 1.218 | 0.708 |
| **LB** | Agriculture | 0.86 ± 0.19 | 1.16 ± 0.27 | 0.739 | 0.336 |
|  | Palm grove | 0.47 ± 0.17 | 0.74 ± 0.15 | 0.639 | 0.27 |

**Table S4.** Pairwise comparisons among sampling months based on estimated marginal means for erythrocytic nuclear abnormalities in adult amphibians from wetlands of southern Brazil. Values are presented as ratios ± standard error (SE). P-values were adjusted using Tukey’s method for multiple comparisons. NAs = total nuclear abnormalities, ENAs = erythrocytic nuclear abnormalities excluding micronuclei, MN = micronuclei, BL = blebbed nuclei, NT = notched nuclei, KS = kidney-shaped nuclei. Significant comparisons (*p* < 0.05) are shown in bold.

| **Biomarker** | **Contrast** | **Ratio** | **SE** | **z-ratio** | ***p*-value** |
| --- | --- | --- | --- | --- | --- |
| **NAs** | December / November | 1.232 | 0.170 | 1.517 | 0.4269 |
|  | December / October | 0.942 | 0.129 | -0.433 | 0.9728 |
|  | December / September | 0.680 | 0.114 | -2.303 | 0.0973 |
|  | November / October | 0.765 | 0.087 | -2.369 | 0.0831 |
|  | November / September | 0.552 | 0.082 | -4.007 | **0.0004** |
|  | October / September | 0.722 | 0.107 | -2.206 | 0.1215 |
| **MN** | December / November | 1.514 | 0.237 | 2.649 | **0.0403** |
|  | December / October | 0.991 | 0.153 | -0.060 | 0.9999 |
|  | December / September | 0.650 | 0.123 | -2.278 | 0.1031 |
|  | November / October | 0.654 | 0.085 | -3.278 | **0.0058** |
|  | November / September | 0.429 | 0.072 | -5.023 | **<0.001** |
|  | October / September | 0.656 | 0.110 | -2.522 | 0.0566 |
| **BL** | December / November | 0.423 | 0.133 | -2.745 | **0.0308** |
|  | December / October | 0.427 | 0.134 | -2.710 | **0.0340** |
|  | December / September | 0.410 | 0.152 | -2.397 | 0.0776 |
|  | November / October | 1.010 | 0.235 | 0.041 | 1.0000 |
|  | November / September | 0.969 | 0.297 | -0.104 | 0.9996 |
|  | October / September | 0.960 | 0.295 | -0.135 | 0.9991 |
| **KS** | December / November | 2.474 | 1.030 | 2.183 | 0.1278 |
|  | December / October | 1.494 | 0.588 | 1.019 | 0.7383 |
|  | December / September | 0.666 | 0.295 | -0.917 | 0.7956 |
|  | November / October | 0.604 | 0.226 | -1.347 | 0.5328 |
|  | November / September | 0.269 | 0.114 | -3.094 | **0.0107** |
|  | October / September | 0.446 | 0.179 | -2.016 | 0.1821 |

**Table S5.** Comparisons between species within each month. Mean (± SD) frequency of nuclear abnormalities in *Dendropsophus sanborni* and *Pseudis minuta* for each biomarker (NAs, ENAs, MN, BL, NT, KS, LB) across sampling months (September–December). NAs = total nuclear abnormalities, ENAs = erythrocytic nuclear abnormalities excluding micronuclei, MN = micronuclei, BL = blebbed nuclei, NT = notched nuclei, and KS = kidney-shaped nuclei. The ratio (*D. sanborni* / *P. minuta*) represents the relative difference between species. *p*-values indicate results of statistical comparisons between species within each month. Significant differences (*p* < 0.05) are indicated with an asterisk.

| **Month** | **Biomarker** | ***D. sanborni*** | ***P. minuta*** | **Ratio** | ***p*-value** |
| --- | --- | --- | --- | --- | --- |
| **September** | NAs | 23.98 ± 5.52 | 26.65 ± 3.86 | 0.90 | 0.698 |
|  | ENA | 4.66 ± 1.85 | 8.29 ± 2.01 | 0.56 | 0.217 |
|  | MN | 19.66 ± 5.06 | 18.55 ± 3.03 | 1.06 | 0.849 |
|  | BL | 0.80 ± 0.45 | 3.29 ± 0.95 | **0.24** | **0.025** |
|  | NT | 1.25 ± 0.60 | 3.76 ± 0.98 | **0.33** | **0.044** |
|  | KS | 1.17 ± 0.59 | 0.62 ± 0.24 | 1.87 | 0.327 |
|  | LB | 1.31 ± 0.64 | 0.46 ± 0.19 | 2.86 | 0.103 |
| **October** | NAs | 15.52 ± 1.81 | 19.72 ± 2.10 | 0.79 | 0.133 |
|  | ENA | 4.14 ± 0.83 | 7.14 ± 1.27 | **0.58** | **0.043** |
|  | MN | 11.31 ± 1.49 | 12.53 ± 1.52 | 0.90 | 0.570 |
|  | BL | 1.09 ± 0.29 | 3.30 ± 0.69 | **0.33** | **0.001** |
|  | NT | 1.86 ± 0.42 | 2.85 ± 0.55 | 0.66 | 0.157 |
|  | KS | 0.48 ± 0.16 | 0.24 ± 0.09 | 2.02 | 0.161 |
|  | LB | 0.78 ± 0.22 | 0.81 ± 0.21 | 0.96 | 0.923 |
| **November** | NAs | 9.61 ± 1.16 | 17.85 ± 1.91 | **0.54** | **<0.001** |
|  | ENA | 3.90 ± 0.77 | 7.69 ± 1.36 | **0.51** | **0.010** |
|  | MN | 5.63 ± 0.79 | 10.16 ± 1.25 | **0.55** | **0.002** |
|  | BL | 1.51 ± 0.37 | 2.68 ± 0.57 | 0.56 | 0.077 |
|  | NT | 1.97 ± 0.43 | 3.41 ± 0.65 | 0.58 | 0.058 |
|  | KS | 0.08 ± 0.05 | 0.34 ± 0.11 | **0.24** | **0.024** |
|  | LB | 0.33 ± 0.11 | 1.21 ± 0.28 | **0.27** | **0.001** |
| **December** | NAs | 15.33 ± 2.65 | 17.87 ± 2.76 | 0.86 | 0.525 |
|  | ENA | 3.93 ± 1.15 | 5.58 ± 1.45 | 0.70 | 0.390 |
|  | MN | 11.61 ± 2.26 | 12.07 ± 2.11 | 0.96 | 0.887 |
|  | BL | 0.96 ± 0.37 | 0.72 ± 0.28 | 1.35 | 0.602 |
|  | NT | 1.63 ± 0.53 | 3.35 ± 0.93 | 0.49 | 0.105 |
|  | KS | 0.47 ± 0.20 | 0.58 ± 0.26 | 0.81 | 0.748 |
|  | LB | 0.75 ± 0.29 | 1.08 ± 0.39 | 0.69 | 0.502 |


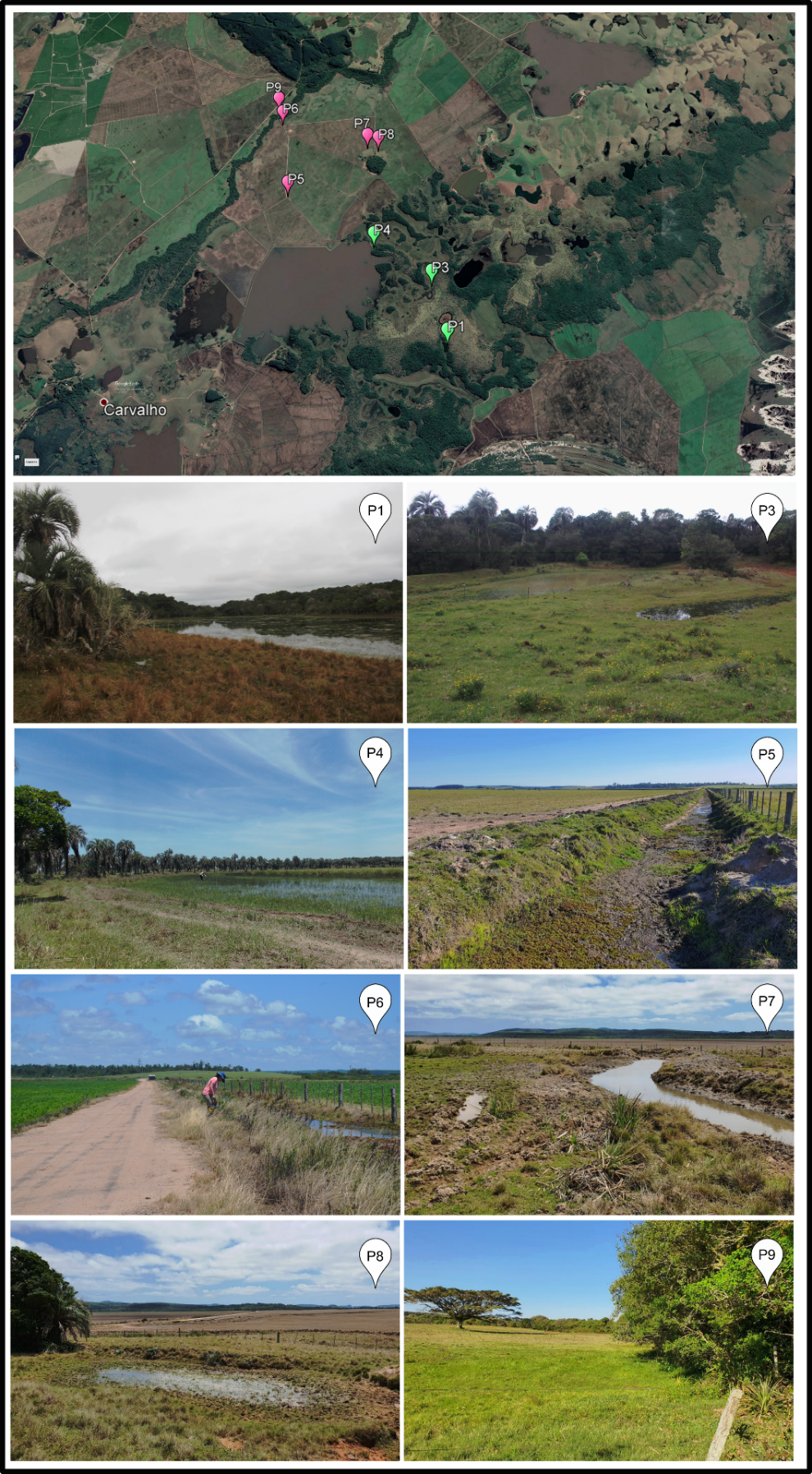


**Fig. S1.** Breeding sites sampled in the study area. P1, P3, and P4 correspond to palm grove (natural) habitats, whereas P5, P6, P7, P8, and P9 correspond to agricultural environments.


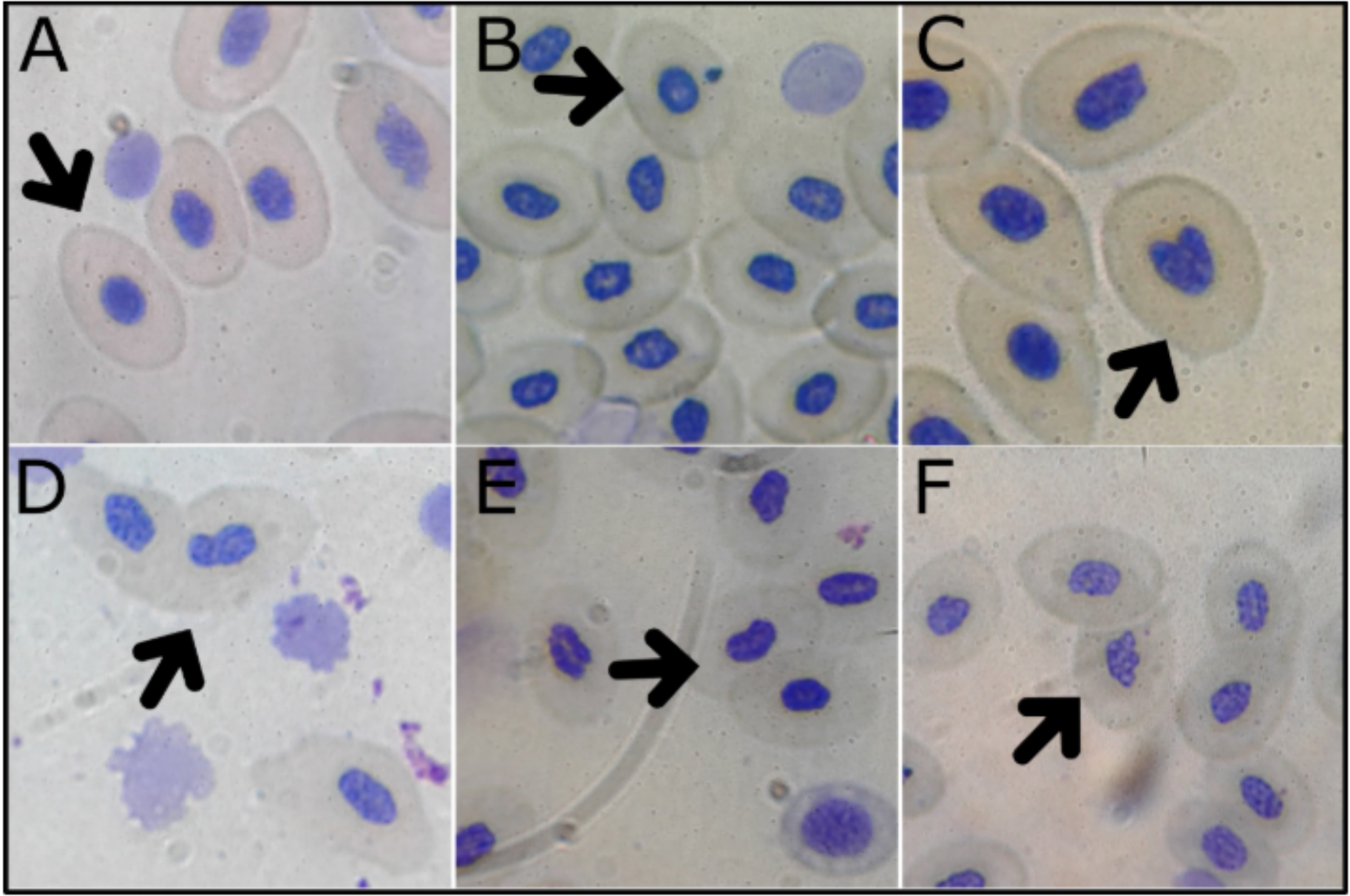


**Fig. S2.** Representative photomicrographs of erythrocytes showing different types of nuclear morphology and abnormalities in amphibians. Panels represent: (A) normal nucleus, (B) micronucleus (MN), (C) notched nucleus (NT), (D) lobed nucleus (LB), (E) kidney-shaped nucleus (KS), and (F) blebbed nucleus (BL). Arrows indicate the structures of interest. Slides were analyzed using a light microscope at 1000× magnification.

**
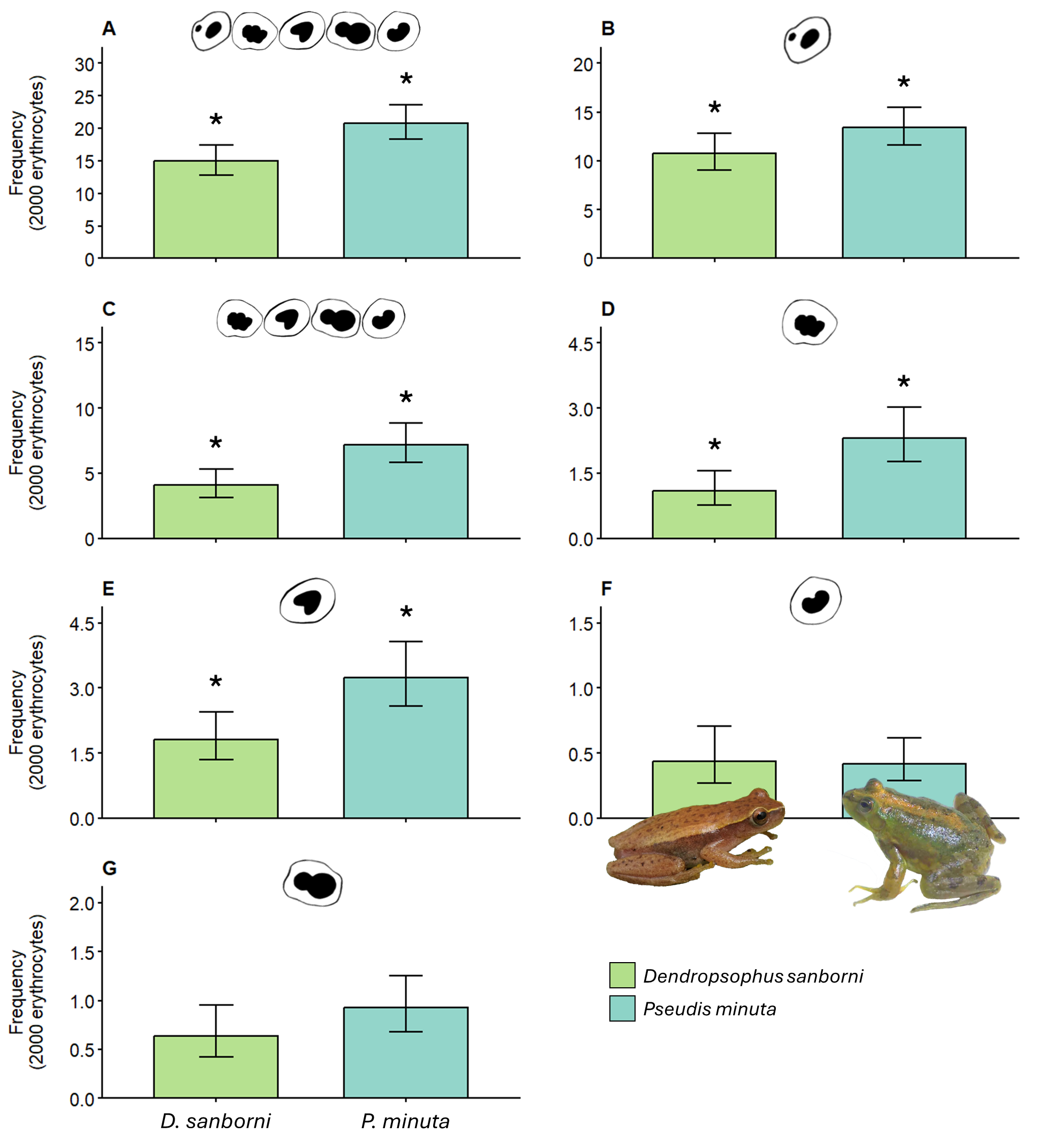
**

**Fig. S3.** Mean (± SD) frequency of erythrocytic nuclear abnormalities in *Dendropsophus sanborni* (green) and *Pseudis minuta* (blue) across all sampled individuals. Panels represent: (A) total nuclear abnormalities (NAs), (B) micronuclei (MN), (C) nuclear abnormalities excluding MN (ENAs), (D) blebbed nuclei (BL), (E) notched nuclei (NT), (F) kidney-shaped nuclei (KS), and (G) lobed nuclei (LB). Frequencies were calculated based on 2,000 erythrocytes per individual. Asterisks indicate significant differences between species (*p* < 0.05).
